# Supplementary material for: Resolving the full spectrum of human genome variation using Linked-Reads
Source: Genome Res. 2019 Apr;29(4):635–45. doi: 10.1101/gr.234443.118 (PMC6442396; doi:10.1101/gr.234443.118)
Supplement: Supplemental Material [file supp_gr.234443.118_Supplemental_longranger-2.2.zip › longranger-2.2.tar.bz2!/lr2.2-public/lib/stan/cmdstan/stan/doc/api/html/bernoulli_8hpp_source.html]

Stan: src/stan/prob/distributions/univariate/discrete/bernoulli.hpp Source File


|  |  |
| --- | --- |
|  | Stan  2.5.0  probability, sampling & optimization |


- Main Page
- Related Pages
- Namespaces
- Classes
- Files

- File List
- File Members

All Classes Namespaces Files Functions Variables Typedefs Enumerations Enumerator Friends Macros Pages

- src
- stan
- prob
- distributions
- univariate
- discrete

bernoulli.hpp

Go to the documentation of this file.

1 #ifndef STAN\_\_PROB\_\_DISTRIBUTIONS\_\_UNIVARIATE\_\_DISCRETE\_\_BERNOULLI\_HPP

2 #define STAN\_\_PROB\_\_DISTRIBUTIONS\_\_UNIVARIATE\_\_DISCRETE\_\_BERNOULLI\_HPP

3

4 #include <boost/random/bernoulli\_distribution.hpp>

5 #include <boost/random/variate\_generator.hpp>

6

7 #include <stan/agrad/partials\_vari.hpp>

8 #include <stan/math/error\_handling.hpp>

9 #include <stan/math/constants.hpp>

10 #include <stan/math/functions/inv\_logit.hpp>

11 #include <stan/math/functions/log1m.hpp>

12 #include <stan/math/functions/value\_of.hpp>

13 #include <stan/meta/traits.hpp>

14 #include <stan/prob/traits.hpp>

15 #include <stan/prob/constants.hpp>

16

17 namespace stan {

18

19  namespace prob {

20

21  // Bernoulli(n|theta) [0 <= n <= 1; 0 <= theta <= 1]

22  // FIXME: documentation

23  template <bool propto, typename T\_n, typename T\_prob>

24  typename return\_type<T\_prob>::type

25  bernoulli\_log(const T\_n& n,

26  const T\_prob& theta) {

27  static const char\* function = "stan::prob::bernoulli\_log(%1%)";

28

29  using stan::math::check\_finite;

30  using stan::math::check\_bounded;

31  using stan::math::log1m;

32  using stan::math::value\_of;

33  using stan::math::check\_consistent\_sizes;

34  using stan::prob::include\_summand;

35

36  // check if any vectors are zero length

37  if (!(stan::length(n)

38  && stan::length(theta)))

39  return 0.0;

40

41  // set up return value accumulator

42  double logp(0.0);

43

44  // validate args (here done over var, which should be OK)

45  check\_bounded(function, n, 0, 1, "n", &logp);

46  check\_finite(function, theta, "Probability parameter", &logp);

47  check\_bounded(function, theta, 0.0, 1.0,

48  "Probability parameter", &logp);

49  check\_consistent\_sizes(function,

50  n,theta,

51  "Random variable","Probability parameter",

52  &logp);

53

54  // check if no variables are involved and prop-to

55  if (!include\_summand<propto,T\_prob>::value)

56  return 0.0;

57

58  // set up template expressions wrapping scalars into vector views

59  VectorView<const T\_n> n\_vec(n);

60  VectorView<const T\_prob> theta\_vec(theta);

61  size\_t N = max\_size(n, theta);

62  agrad::OperandsAndPartials<T\_prob> operands\_and\_partials(theta);

63

64  if (length(theta) == 1) {

65  size\_t sum = 0;

66  for (size\_t n = 0; n < N; n++) {

67  sum += value\_of(n\_vec[n]);

68  }

69  const double theta\_dbl = value\_of(theta\_vec[0]);

70  // avoid nans when sum == N or sum == 0

71  if (sum == N) {

72  logp += N \* log(theta\_dbl);

73  if (!is\_constant\_struct<T\_prob>::value)

74  operands\_and\_partials.d\_x1[0] += N / theta\_dbl;

75  } else if (sum == 0) {

76  logp += N \* log1m(theta\_dbl);

77  if (!is\_constant\_struct<T\_prob>::value)

78  operands\_and\_partials.d\_x1[0] += N / (theta\_dbl - 1);

79  } else {

80  const double log\_theta = log(theta\_dbl);

81  const double log1m\_theta = log1m(theta\_dbl);

82

83  logp += sum \* log\_theta;

84  logp += (N - sum) \* log1m\_theta;

85

86  // gradient

87  if (!is\_constant\_struct<T\_prob>::value) {

88  operands\_and\_partials.d\_x1[0] += sum / theta\_dbl;

89  operands\_and\_partials.d\_x1[0] += (N - sum) / (theta\_dbl - 1);

90  }

91  }

92  } else {

93  for (size\_t n = 0; n < N; n++) {

94  // pull out values of arguments

95  const int n\_int = value\_of(n\_vec[n]);

96  const double theta\_dbl = value\_of(theta\_vec[n]);

97

98  if (n\_int == 1)

99  logp += log(theta\_dbl);

100  else

101  logp += log1m(theta\_dbl);

102

103  // gradient

104  if (!is\_constant\_struct<T\_prob>::value) {

105  if (n\_int == 1)

106  operands\_and\_partials.d\_x1[n] += 1.0 / theta\_dbl;

107  else

108  operands\_and\_partials.d\_x1[n] += 1.0 / (theta\_dbl - 1);

109  }

110  }

111  }

112  return operands\_and\_partials.to\_var(logp);

113  }

114

115  template <typename T\_y, typename T\_prob>

116  inline

117  typename return\_type<T\_prob>::type

118  bernoulli\_log(const T\_y& n,

119  const T\_prob& theta) {

120  return bernoulli\_log<false>(n,theta);

121  }

122

123

124  // Bernoulli(n|inv\_logit(theta)) [0 <= n <= 1; -inf <= theta <= inf]

125  // FIXME: documentation

126  template <bool propto, typename T\_n, typename T\_prob>

127  typename return\_type<T\_prob>::type

128  bernoulli\_logit\_log(const T\_n& n, const T\_prob& theta) {

129  static const char\* function = "stan::prob::bernoulli\_logit\_log(%1%)";

130

131  using stan::is\_constant\_struct;

132  using stan::math::check\_not\_nan;

133  using stan::math::check\_bounded;

134  using stan::math::value\_of;

135  using stan::math::check\_consistent\_sizes;

136  using stan::prob::include\_summand;

137  using stan::math::log1p;

138  using stan::math::inv\_logit;

139

140  // check if any vectors are zero length

141  if (!(stan::length(n)

142  && stan::length(theta)))

143  return 0.0;

144

145  // set up return value accumulator

146  double logp(0.0);

147

148  // validate args (here done over var, which should be OK)

149  check\_bounded(function, n, 0, 1, "n", &logp);

150  check\_not\_nan(function, theta, "Logit transformed probability parameter",

151  &logp);

152  check\_consistent\_sizes(function,

153  n,theta,

154  "Random variable","Probability parameter",

155  &logp);

156

157  // check if no variables are involved and prop-to

158  if (!include\_summand<propto,T\_prob>::value)

159  return 0.0;

160

161  // set up template expressions wrapping scalars into vector views

162  VectorView<const T\_n> n\_vec(n);

163  VectorView<const T\_prob> theta\_vec(theta);

164  size\_t N = max\_size(n, theta);

165  agrad::OperandsAndPartials<T\_prob> operands\_and\_partials(theta);

166

167  for (size\_t n = 0; n < N; n++) {

168  // pull out values of arguments

169  const int n\_int = value\_of(n\_vec[n]);

170  const double theta\_dbl = value\_of(theta\_vec[n]);

171

172  // reusable subexpression values

173  const int sign = 2\*n\_int-1;

174  const double ntheta = sign \* theta\_dbl;

175  const double exp\_m\_ntheta = exp(-ntheta);

176

177  // Handle extreme values gracefully using Taylor approximations.

178  const static double cutoff = 20.0;

179  if (ntheta > cutoff)

180  logp -= exp\_m\_ntheta;

181  else if (ntheta < -cutoff)

182  logp += ntheta;

183  else

184  logp -= log1p(exp\_m\_ntheta);

185

186  // gradients

187  if (!is\_constant\_struct<T\_prob>::value) {

188  const static double cutoff = 20.0;

189  if (ntheta > cutoff)

190  operands\_and\_partials.d\_x1[n] -= exp\_m\_ntheta;

191  else if (ntheta < -cutoff)

192  operands\_and\_partials.d\_x1[n] += sign;

193  else

194  operands\_and\_partials.d\_x1[n] += sign \* exp\_m\_ntheta / (exp\_m\_ntheta + 1);

195  }

196  }

197  return operands\_and\_partials.to\_var(logp);

198  }

199

200  template <typename T\_n,

201  typename T\_prob>

202  inline

203  typename return\_type<T\_prob>::type

204  bernoulli\_logit\_log(const T\_n& n,

205  const T\_prob& theta) {

206  return bernoulli\_logit\_log<false>(n,theta);

207  }

208

209  // Bernoulli CDF

210  template <typename T\_n, typename T\_prob>

211  typename return\_type<T\_prob>::type

212  bernoulli\_cdf(const T\_n& n, const T\_prob& theta) {

213  static const char\* function = "stan::prob::bernoulli\_cdf(%1%)";

214

215  using stan::math::check\_finite;

216  using stan::math::check\_bounded;

217  using stan::math::check\_consistent\_sizes;

218  using stan::prob::include\_summand;

219

220  // Ensure non-zero argument lenghts

221  if (!(stan::length(n) && stan::length(theta)))

222  return 1.0;

223

224  double P(1.0);

225

226  // Validate arguments

227  check\_finite(function, theta, "Probability parameter", &P);

228  check\_bounded(function, theta, 0.0, 1.0,

229  "Probability parameter", &P);

230  check\_consistent\_sizes(function,

231  n, theta,

232  "Random variable","Probability parameter",

233  &P);

234

235  // set up template expressions wrapping scalars into vector views

236  VectorView<const T\_n> n\_vec(n);

237  VectorView<const T\_prob> theta\_vec(theta);

238  size\_t size = max\_size(n, theta);

239

240  // Compute vectorized CDF and gradient

241  using stan::math::value\_of;

242  agrad::OperandsAndPartials<T\_prob> operands\_and\_partials(theta);

243

244  // Explicit return for extreme values

245  // The gradients are technically ill-defined, but treated as zero

246  for (size\_t i = 0; i < stan::length(n); i++) {

247  if (value\_of(n\_vec[i]) < 0)

248  return operands\_and\_partials.to\_var(0.0);

249  }

250

251  for (size\_t i = 0; i < size; i++) {

252

253  // Explicit results for extreme values

254  // The gradients are technically ill-defined, but treated as zero

255  if (value\_of(n\_vec[i]) >= 1) continue;

256  else {

257  const double Pi = 1 - value\_of(theta\_vec[i]);

258

259  P \*= Pi;

260

261  if (!is\_constant\_struct<T\_prob>::value)

262  operands\_and\_partials.d\_x1[i] += - 1 / Pi;

263  }

264  }

265

266  if (!is\_constant\_struct<T\_prob>::value) {

267  for(size\_t i = 0; i < stan::length(theta); ++i) operands\_and\_partials.d\_x1[i] \*= P;

268  }

269  return operands\_and\_partials.to\_var(P);

270  }

271

272  template <typename T\_n, typename T\_prob>

273  typename return\_type<T\_prob>::type

274  bernoulli\_cdf\_log(const T\_n& n, const T\_prob& theta) {

275  static const char\* function = "stan::prob::bernoulli\_cdf\_log(%1%)";

276

277  using stan::math::check\_finite;

278  using stan::math::check\_bounded;

279  using stan::math::check\_consistent\_sizes;

280  using stan::prob::include\_summand;

281

282  // Ensure non-zero argument lenghts

283  if (!(stan::length(n) && stan::length(theta)))

284  return 0.0;

285

286  double P(0.0);

287

288  // Validate arguments

289  check\_finite(function, theta, "Probability parameter", &P);

290  check\_bounded(function, theta, 0.0, 1.0,

291  "Probability parameter", &P);

292  check\_consistent\_sizes(function,

293  n, theta,

294  "Random variable","Probability parameter",

295  &P);

296

297  // set up template expressions wrapping scalars into vector views

298  VectorView<const T\_n> n\_vec(n);

299  VectorView<const T\_prob> theta\_vec(theta);

300  size\_t size = max\_size(n, theta);

301

302  // Compute vectorized cdf\_log and gradient

303  using stan::math::value\_of;

304  agrad::OperandsAndPartials<T\_prob> operands\_and\_partials(theta);

305

306  // Explicit return for extreme values

307  // The gradients are technically ill-defined, but treated as zero

308  for (size\_t i = 0; i < stan::length(n); i++) {

309  if (value\_of(n\_vec[i]) < 0)

310  return operands\_and\_partials.to\_var(stan::math::negative\_infinity());

311  }

312

313  for (size\_t i = 0; i < size; i++) {

314

315  // Explicit results for extreme values

316  // The gradients are technically ill-defined, but treated as zero

317  if (value\_of(n\_vec[i]) >= 1) continue;

318  else {

319  const double Pi = 1 - value\_of(theta\_vec[i]);

320

321  P += log(Pi);

322

323  if (!is\_constant\_struct<T\_prob>::value)

324  operands\_and\_partials.d\_x1[i] -= 1 / Pi;

325  }

326  }

327

328  return operands\_and\_partials.to\_var(P);

329  }

330

331  template <typename T\_n, typename T\_prob>

332  typename return\_type<T\_prob>::type

333  bernoulli\_ccdf\_log(const T\_n& n, const T\_prob& theta) {

334  static const char\* function = "stan::prob::bernoulli\_ccdf\_log(%1%)";

335

336  using stan::math::check\_finite;

337  using stan::math::check\_bounded;

338  using stan::math::check\_consistent\_sizes;

339  using stan::prob::include\_summand;

340

341  // Ensure non-zero argument lenghts

342  if (!(stan::length(n) && stan::length(theta)))

343  return 0.0;

344

345  double P(0.0);

346

347  // Validate arguments

348  check\_finite(function, theta, "Probability parameter", &P);

349  check\_bounded(function, theta, 0.0, 1.0,

350  "Probability parameter", &P);

351  check\_consistent\_sizes(function,

352  n, theta,

353  "Random variable","Probability parameter",

354  &P);

355

356  // set up template expressions wrapping scalars into vector views

357  VectorView<const T\_n> n\_vec(n);

358  VectorView<const T\_prob> theta\_vec(theta);

359  size\_t size = max\_size(n, theta);

360

361  // Compute vectorized cdf\_log and gradient

362  using stan::math::value\_of;

363  agrad::OperandsAndPartials<T\_prob> operands\_and\_partials(theta);

364

365  // Explicit return for extreme values

366  // The gradients are technically ill-defined, but treated as zero

367  for (size\_t i = 0; i < stan::length(n); i++) {

368  if (value\_of(n\_vec[i]) < 0)

369  return operands\_and\_partials.to\_var(0.0);

370  }

371

372  for (size\_t i = 0; i < size; i++) {

373

374  // Explicit results for extreme values

375  // The gradients are technically ill-defined, but treated as zero

376  if (value\_of(n\_vec[i]) >= 1)

377  return operands\_and\_partials.to\_var(stan::math::negative\_infinity());

378  else {

379  const double Pi = value\_of(theta\_vec[i]);

380

381  P += log(Pi);

382

383  if (!is\_constant\_struct<T\_prob>::value)

384  operands\_and\_partials.d\_x1[i] += 1 / Pi;

385  }

386  }

387

388  return operands\_and\_partials.to\_var(P);

389  }

390

391

392  template <class RNG>

393  inline int

394  bernoulli\_rng(const double theta,

395  RNG& rng) {

396  using boost::variate\_generator;

397  using boost::bernoulli\_distribution;

398

399  static const char\* function = "stan::prob::bernoulli\_rng(%1%)";

400

401  using stan::math::check\_finite;

402  using stan::math::check\_bounded;

403

404  check\_finite(function, theta, "Probability parameter", (double\*)0);

405  check\_bounded(function, theta, 0, 1,

406  "Probability parameter", (double\*)0);

407

408  variate\_generator<RNG&, bernoulli\_distribution<> >

409  bernoulli\_rng(rng, bernoulli\_distribution<>(theta));

410  return bernoulli\_rng();

411  }

412  }

413 }

414 #endif

stan::agrad::log1m

fvar< T > log1m(const fvar< T > &x)

**Definition:** log1m.hpp:16

stan::math::check\_bounded

bool check\_bounded(const char \*function, const T\_y &y, const T\_low &low, const T\_high &high, const char \*name, T\_result \*result)

**Definition:** check\_bounded.hpp:79

traits.hpp

stan::math::log1p

boost::math::tools::promote\_args< T >::type log1p(const T x)

Return the natural logarithm of one plus the specified value.

**Definition:** log1p.hpp:39

stan::agrad::OperandsAndPartials::to\_var

T\_return\_type to\_var(double logp)

**Definition:** partials\_vari.hpp:138

stan::math::sign

int sign(const T &z)

**Definition:** sign.hpp:9

error\_handling.hpp

stan::length

size\_t length(const T &)

**Definition:** traits.hpp:159

stan::math::check\_finite

bool check\_finite(const char \*function, const T\_y &y, const char \*name, T\_result \*result)

Checks if the variable y is finite.

**Definition:** check\_finite.hpp:52

stan::math::inv\_logit

boost::math::tools::promote\_args< T >::type inv\_logit(const T a)

Returns the inverse logit function applied to the argument.

**Definition:** inv\_logit.hpp:52

stan::prob::bernoulli\_ccdf\_log

return\_type< T\_prob >::type bernoulli\_ccdf\_log(const T\_n &n, const T\_prob &theta)

**Definition:** bernoulli.hpp:333

stan::prob::bernoulli\_rng

int bernoulli\_rng(const double theta, RNG &rng)

**Definition:** bernoulli.hpp:394

partials\_vari.hpp

stan::agrad::value\_of

T value\_of(const fvar< T > &v)

Return the value of the specified variable.

**Definition:** value\_of.hpp:16

constants.hpp

stan::agrad::sum

fvar< T > sum(const Eigen::Matrix< fvar< T >, R, C > &m)

**Definition:** sum.hpp:14

stan::agrad::OperandsAndPartials

A variable implementation that stores operands and derivatives with respect to the variable...

**Definition:** partials\_vari.hpp:76

traits.hpp

stan::return\_type::type

boost::math::tools::promote\_args< typename scalar\_type< T1 >::type, typename scalar\_type< T2 >::type, typename scalar\_type< T3 >::type, typename scalar\_type< T4 >::type, typename scalar\_type< T5 >::type, typename scalar\_type< T6 >::type >::type type

**Definition:** traits.hpp:406

stan::is\_constant\_struct

Metaprogram to determine if a type has a base scalar type that can be assigned to type double...

**Definition:** traits.hpp:57

stan::math::value\_of

double value\_of(const T x)

Return the value of the specified scalar argument converted to a double value.

**Definition:** value\_of.hpp:24

stan::prob::include\_summand

Template metaprogram to calculate whether a summand needs to be included in a proportional (log) prob...

**Definition:** traits.hpp:35

log1m.hpp

stan::math::check\_consistent\_sizes

bool check\_consistent\_sizes(const char \*function, const T1 &x1, const T2 &x2, const char \*name1, const char \*name2, T\_result \*result)

**Definition:** check\_consistent\_sizes.hpp:13

stan::max\_size

size\_t max\_size(const T1 &x1, const T2 &x2)

**Definition:** traits.hpp:191

stan::prob::bernoulli\_log

return\_type< T\_prob >::type bernoulli\_log(const T\_n &n, const T\_prob &theta)

**Definition:** bernoulli.hpp:25

stan::prob::bernoulli\_cdf\_log

return\_type< T\_prob >::type bernoulli\_cdf\_log(const T\_n &n, const T\_prob &theta)

**Definition:** bernoulli.hpp:274

stan::math::check\_not\_nan

bool check\_not\_nan(const char \*function, const T\_y &y, const char \*name, T\_result \*result)

Checks if the variable y is nan.

**Definition:** check\_not\_nan.hpp:56

stan::math::size

int size(const std::vector< T > &x)

**Definition:** size.hpp:11

stan::prob::bernoulli\_cdf

return\_type< T\_prob >::type bernoulli\_cdf(const T\_n &n, const T\_prob &theta)

**Definition:** bernoulli.hpp:212

value\_of.hpp

inv\_logit.hpp

stan::agrad::OperandsAndPartials::d\_x1

VectorView< double \*, is\_vector< T1 >::value, is\_constant\_struct< T1 >::value > d\_x1

**Definition:** partials\_vari.hpp:82

constants.hpp

stan::agrad::log

fvar< T > log(const fvar< T > &x)

**Definition:** log.hpp:15

stan::prob::bernoulli\_logit\_log

return\_type< T\_prob >::type bernoulli\_logit\_log(const T\_n &n, const T\_prob &theta)

**Definition:** bernoulli.hpp:128

stan::VectorView

VectorView is a template metaprogram that takes its argument and allows it to be used like a vector...

**Definition:** traits.hpp:275

stan::agrad::exp

fvar< T > exp(const fvar< T > &x)

**Definition:** exp.hpp:16

stan::agrad::log1p

fvar< T > log1p(const fvar< T > &x)

**Definition:** log1p.hpp:16

stan::math::log1m

boost::math::tools::promote\_args< T >::type log1m(T x)

Return the natural logarithm of one minus the specified value.

**Definition:** log1m.hpp:40

stan::math::negative\_infinity

double negative\_infinity()

Return negative infinity.

**Definition:** constants.hpp:123


---

[ Stan Home Page ]

*© 2011–2014,
Stan Development Team.*
